# Unrecognized Inter-Coronary Communication in a Case of Hypertrophic Cardiomyopathy

**DOI:** 10.15190/d.2024.10

**Published:** 2024-09-30

**Authors:** Khalid W. Al-Kaissi, Arpita Meher, Ayeza Majid, Yamen Hussein

**Affiliations:** ^1^Department of Cardiology, Rashid Hospital, Dubai, United Arab Emirates; ^2^Department of Medicine, Tbilisi State Medical University, Tbilisi, Georgia

**Keywords:** Intercoronary communication, coronary artery anomaly, hypertrophic obstructive cardiomyopathy

## Abstract

Inter-coronary communication is a rare congenital anomaly, defined as a connection between two patent coronary arteries, and was first described in 1972. We report the case of a 61-year-old Emirati female who presented to the emergency department with chest pain and palpitations, along with a strong family history of cardiac disease. She was initially diagnosed with indolent hypertrophic obstructive cardiomyopathy and accordingly managed; however, the patient remained symptomatic. Further investigations revealed inter-coronary communication between her left circumflex (LCX) and left anterior descending (LAD) coronary arteries. This case highlights the importance of recognizing inter-coronary communications for better outcomes in patients with HOCM, as well as their potential clinical significance with the need for further studies to be done to pinpoint the true significance.

## INTRODUCTION

Coronary artery anomalies are deviations from the normal pattern of coronary circulation. Intercoronary communication (ICC) is a rare congenital anomaly first described in 1972 ^^1^^. The incidence of coronary artery anomalies detected during routine coronary angiography is estimated to be between 0.5% and 1.0%. ICC is an infrequent occurrence among these anomalies, with an incidence as low as 0.002% ^^2,3^^. This anomaly is thought to result from a defect in embryological development that allows these intercoronary channels to persist.

Most coronary artery anomalies remain asymptomatic until later in life and are often discovered incidentally during diagnostic procedures or postmortem examinations, however in a handful of individuals they tend to cause ischemia and worsen pre-existing conditions. Large ICCs without obstructive coronary artery disease are considered infrequent congenital anomalies and are typically found in distinct anatomical locations ^^4^^.

## CASE PRESENTATION

This case involves a 61-year-old female who is pre-diabetic with an HbA1c of 6% (Table 1). She presented with chest pain and palpitations, reporting central, non-crushing chest pain associated with exertion. Her family history is notable for myocardial infarction in her uncles and a pacemaker in her father. On presentation, her blood pressure was 106/69 mmHg, pulse rate was 86 beats per minute, oxygen saturation was 99% on room air, body temperature was 36.5°C, and respiratory rate was 18 breaths per minute. Her cardiopulmonary examination was unremarkable. The table below shows the lab findings on presentation.

**Table 1:** Table 1. Lab Findings on presentation

Test	Value	Unit	Reference Range
Hemoglobin	13.2	g/dl	12-15 g/dl
HBa1C	6	%	<5.7%
Troponin-T	12	ng/L	<14 ng/L
CK-MB	5	ng/ml	<4.89ng/ml
LDL Cholesterol	99	mg/dl	<115mg/dl
HDL Cholesterol	70	mg/dl	>48mg/dl
BNP	1406	pg/ml	<125pg/ml

**Figure 1:**
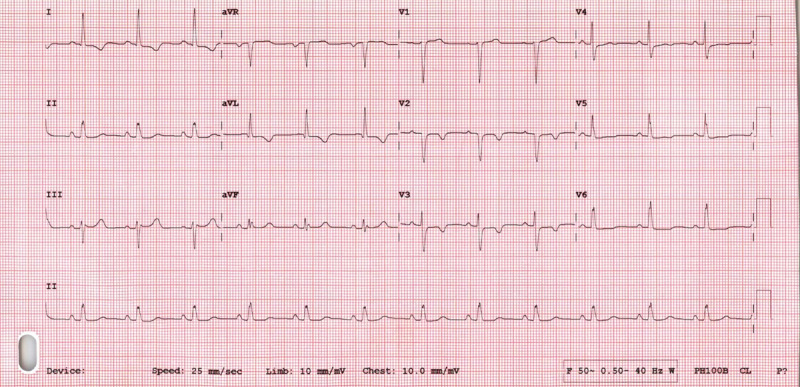
FIgure 1. Resting Electrocardiogram at time of presentation showing normal sinus rhythm with T wave inversions in the lead I, aVL, V2 and V3

Electrocardiogram (ECG) revealed normal sinus rhythm with T wave inversions in lead I, aVL, V2, and V3 (Figure 1). 53 hours Holter ECG revealed normal baseline Sinus Rhythm and unremarkable study.

**Figure 2:**
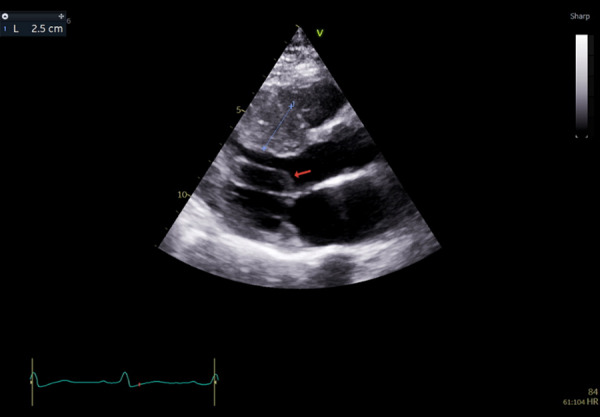
FIgure 2. Long Axis View Parasternal showing Systolic Anterior Motion (Arrow) sign, Hypertrophic Septum Measuring 2.5 cm (blue indicator)

The transthoracic echocardiogram (TTE) (Figure 2) revealed a normal left ventricular internal dimension with severe eccentric left ventricular (LV) hypertrophy. The LV mass was measured at 237 grams, and there was a severely increased left ventricular septal wall thickness, with a basal septal wall thickness of 2.5 cm. The left ventricular systolic function was normal, with an ejection fraction (EF) between 60-65%. There was severe systolic anterior motion (SAM) of the mitral valve, with a peak left ventricular outflow tract (LVOT) pressure gradient increasing from 16 mmHg to 89 mmHg during the Valsalva maneuver. The diastolic filling pattern indicated impaired relaxation. These echocardiographic findings were suggestive of hypertrophic obstructive cardiomyopathy (HOCM).

**Figure 3:**
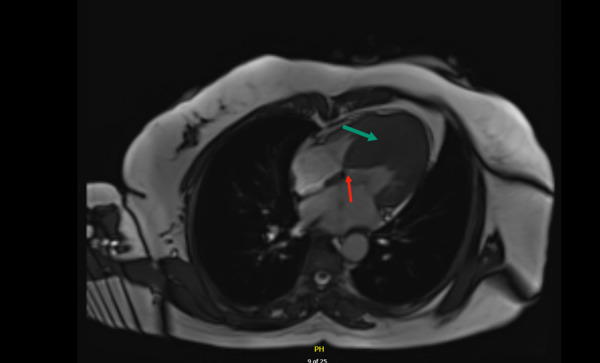
FIgure 3. Magnetic Resonance Imaging Cranial View 4 Chamber Systolic phase shows obliteration of left ventricular cavity during Systole (green arrow), and chordae systolic anterior motion causing Left Ventricular Outflow tract obstruction (red arrow).

**Figure 4:**
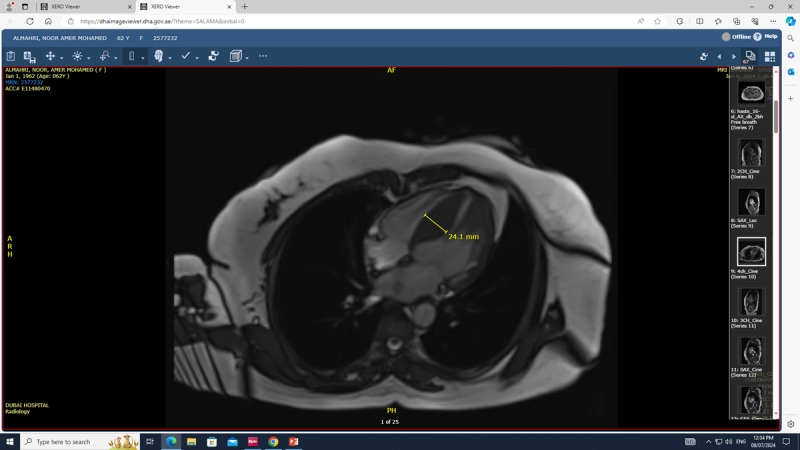
Figure 4. MRI Cranial 4 chamber view diastolic phase showing Hypertrophic Cardiomyopathy with Septum diameter measuring 24.1mm (yellow indicator).

Stress Cardiac Magnetic Resonance Imaging (MRI) revealed left ventricular myocardial hypertrophy, which was more prominent in the septum, with a maximum wall thickness of 2.83 cm. There was complete obliteration of the left ventricular cavity during systole, with systolic anterior motion (SAM) of the chordae causing left ventricular outflow tract (LVOT) obstruction. Additionally, there was diffuse mid-left ventricular wall delayed enhancement and evidence of myocardial scarring. A positive stress test indicated ischemia in the hypertrophic segment, confirming the diagnosis of hypertrophic obstructive cardiomyopathy (HOCM) (Figures 3,4).

**Figure 5:**
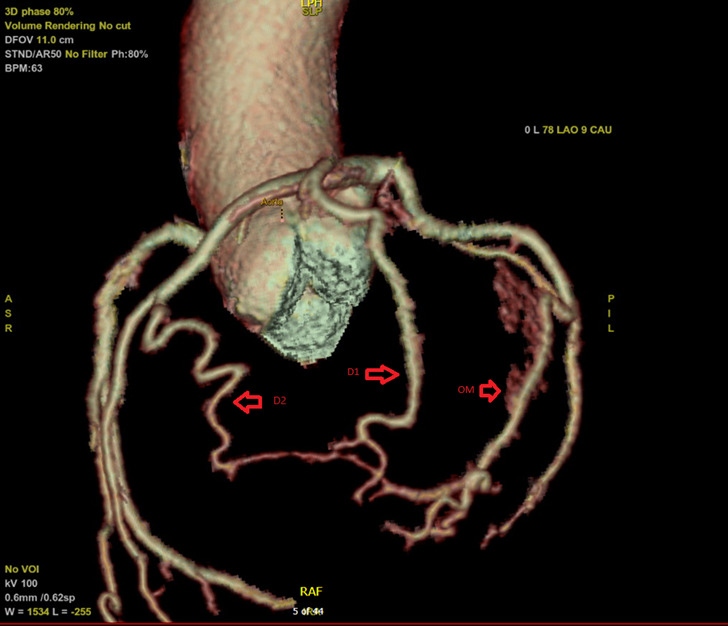
Figure 5. Computed Tomography Angiography showing the connection between the Obtuse Marginal Branch (OM Arrow) of the Left Circumflex Artery with the first Diagonal (D1 Arrow) and second Diagonal branches (D2 Arrow) of the Left Anterior Descending artery.

The patient was initially managed medically with bisoprolol 7.5 mg twice daily and was subsequently discharged. During follow-up, the patient continued to experience symptoms despite medical management. Therefore, a Computed Tomography Angiogram (CTA) was performed, which revealed a possible fistulous communication between the obtuse marginal (OM) branch of the left circumflex (LCX) artery and the Diagonal 1 (D1) and Diagonal 2 (D2) branches of the left anterior descending (LAD) artery (Figure 5). In the end, the patient declined further investigations and was continued on the same medical treatment.

The patient’s decision to decline further investigations and possible treatments was not recommended as the atypical presentation of HOCM in this case was attributed to the abnormal finding of ICC.

## DISCUSSION

This case describes the unusual combination of hypertrophic obstructive cardiomyopathy (HOCM) and pre-diabetes in a 61-year-old female, with novel diagnostic and anatomical findings. Advanced imaging methods, such as stress cardiac MRI and TTE, revealed the patient's substantial left ventricular hypertrophy, systolic anterior motion (SAM), and extensive LVOT blockage. CTA revealed an unusual coronary artery fistula between the obtuse marginal branch of the LCX and the diagonal branches of the LAD, which adds to the intricacy. Despite medical treatment, the patient remained symptomatic, highlighting the need for tailored and potentially new therapeutic techniques in such complex instances.

Intercoronary communication is a rare coronary artery abnormality involving direct contact between two unobstructed coronary arteries, allowing unidirectional or bidirectional blood flow. This abnormality is separate from coronary collaterals, common in individuals with occlusive coronary artery disease. ICCs are often bigger in diameter and straighter than tortuous collateral arteries, with histological structures similar to normal artery walls ^^3^^.

### Types of Inter-Coronary Communication

There are two main types of intercoronary communication: communication between the left circumflex artery (LCX) and right coronary artery (RCA) in the posterior atrioventricular groove, and communication between the left anterior descending artery (LAD) and posterior descending artery in the distal interventricular groove ^^3,5^^.

These connections are congenital and separate from the collateral vessels seen in severe coronary artery disease. ICCs are typically bigger in diameter (>1 mm) and straighter than tortuous collateral vessels^^6^^.

ICCs can have either unidirectional or bidirectional blood flow between the two linked coronary arteries. The connected vessel possesses the histological anatomy of a normal artery wall, including a well-defined muscle layer^^3,5^^.

### Clinical Significance

The therapeutic importance of intercoronary communication is multidimensional, with both protective and harmful effects on the myocardium.

#### Protective Effects

*Myocardial Protection*: In the event of a coronary artery obstruction, the ICC can operate as a protective mechanism by sustaining blood flow to the myocardial. This is especially essential when the blockage is just partial, as the ICC can compensate for the diminished blood flow ^^3^^.

*Reduced Ischemia*: ICC can lower the risk of ischemia by providing an alternate blood supply to the myocardium. This is particularly noticeable when the ICC is bidirectional, allowing blood flow to be shifted between the two linked coronary arteries ^^7^^.

#### Detrimental Effects

*Coronary Steal:* Unidirectional ICC can cause a "coronary steal" effect, in which blood flow is redirected away from a stenotic or blocked coronary artery, aggravating ischemia ^^3,7^^.

*Competitive Blood Flow:* The competitive blood flow dynamics inside the ICC might cause fluctuations in blood flow based on the degree of stenosis in the linked coronary arteries. This can have unexpected and perhaps harmful effects on the myocardium ^^7^^.

### Clinical Implications

The clinical implications of ICC are not entirely known, however numerous possible outcomes have been reported: If a severe coronary artery blockage develops in one of the connected arteries, the ICC may protect the myocardium. The communication might provide an alternate blood supply to the damaged region ^^3^^.

However, ICC can cause myocardial ischemia owing to the "coronary steal" phenomenon, in which blood flows preferentially via the connection, depriving the myocardium of sufficient perfusion. This is more likely to happen with unidirectional ICC.ICC has been linked to spontaneous coronary vasospasm in rare situations. Provocation testing for coronary vasospasm may be beneficial in patients with ICC and chest discomfort, even in the absence of considerable coronary constriction ^^8^^. Large, convoluted ICCs may produce symptoms from blood shunting between the coronary arteries ^^9^^. Direct connections between distal coronary arteries are infrequently seen angiographically, and their clinical relevance is unknown ^^10^^.

### Diagnosis and Treatment 

Intercoronary communication (ICC) is an uncommon congenital cardiac defect that involves a direct connection of two or more coronary arteries. It is distinguishable from collateral vessels by its bigger diameter (>1 mm), straight course, and histological structure, which contains a distinct muscle layer. ICC can be either unidirectional or bidirectional, with blood flowing between the linked arteries. ICC is frequently discovered incidentally during coronary angiography, especially in individuals with no substantial coronary artery disease. Treatment for ICC is often cautious because it does not indicate underlying coronary disease. If ICC is accompanied by obstructive coronary artery disease, percutaneous intervention with a drug-eluting stent may be required to treat the underlying ailment ^^9,10^^.

### Insights Related to Our Case

Anatomical considerations play an important role in hypertrophic obstructive cardiomyopathy. The major blockage in HOCM is caused by the hypertrophied septum and anterior leaflet of the mitral valve, resulting in a dynamic pressure gradient across the left ventricular outflow tract. This impediment can drag the mitral valve towards the septum, resulting in the creation of intercoronary connections. HOCM is also frequently linked with aberrant coronary artery morphology, such as anomalous origins, courses, or insertions. These defects can cause intercoronary connections, complicating HOCM diagnosis and therapy ^^11,12^^.

Genetic factors have a crucial role in hypertrophic obstructive cardiomyopathy (HOCM). Mutations in genes encoding sarcomere proteins, including β-myosin heavy chain (MYH7) and myosin-binding protein C (MYBPC3), are major causes of HOCM. These mutations can impair normal heart function, perhaps resulting in the formation of intercoronary connections. Furthermore, some genetic polymorphisms, such as the D/D genotype of the angiotensin-converting enzyme (ACE) gene, might alter the clinical presentation of HOCM and may help to build intercoronary linkages^13^. Increased left ventricular pressure owing to left ventricular outflow tract obstruction (LVOTO) can cause the coronary arteries to widen and link with one another, resulting in intercoronary connections ^^11^^. Additionally, aberrant coronary blood flow patterns in hypertrophic obstructive cardiomyopathy might aid in the creation of these linkages. The increasing pressure gradient across the LVOT can compress the coronary arteries, changing blood flow patterns and favoring the formation of intercoronary connections ^^12^^.

Hypertrophic obstructive cardiomyopathy is characterized by increased myocardial oxygen demand and stress. The increased pressure and volume overload iraisesM raises myocardial oxygen demand, causing the coronary arteries to dilate and establish connections to meet the increased demand. Furthermore, the pressure and volume overload causes increased myocardial stress, resulting in the creation of intercoronary connections as the coronary arteries adjust to the increased stress ^^12^^.

### Future Insights 

Intercoronary communication in hypertrophic obstructive cardiomyopathy is crucial for understanding and controlling the condition. This communication refers to the link between the left and right ventricular coronary arteries, which is critical for heart function and perfusion. In HOCM, LV outflow tract blockage frequently causes lower coronary flow and an increased risk of ischemia, particularly in the subendocardial layers. Future research focuses on understanding microvascular dysfunction, which is critical for designing successful therapies. By uncovering the underlying processes, researchers hope to develop medicines that improve coronary perfusion and reduce the risk of ischemia ^^14^^.

Furthermore, evaluating coronary flow reserve using modern imaging techniques such as cardiac magnetic resonance imaging (MRI) gives extensive insights into coronary flow and perfusion, which aids in the diagnosis and management of HOCM. Novel therapy techniques, like as pacing and ablation, are also being researched to increase coronary perfusion and minimize ischemia risks in HOCM patients. Furthermore, genetic research is identifying the hereditary roots of HOCM, which might lead to tailored medicines that improve coronary function while lowering the risk of complications ^^15^^.

The importance of this clinical case is as follows:

· The case involves a rare congenital anomaly, inter-coronary communication, which is an unusual connection between coronary arteries. With an incidence as low as 0.002%

· The use of multiple diagnostic modalities—such as TTE, stress cardiac MRI, and CTA—illustrates the importance of a comprehensive approach in evaluating complex cardiac cases.

· The case shows that despite medical management with bisoprolol, the patient remained symptomatic. This scenario demonstrates the challenge of managing HOCM patients.

## CONCLUSION

Intercoronary communications exhibit distinct anatomical and functional characteristics that differentiate them from collateral vessels. Their unique features include a singular, extramural location and a larger diameter, as well as a well-defined muscular layer. Unlike collateral vessels, ICCs typically do not develop as a response to coronary artery disease and show histological differences, such as better-organized collagen and muscle fibers.

Functionally, ICCs can redirect blood flow to myocardial regions at risk or, conversely, potentially induce myocardial ischemia depending on the flow direction. Their larger diameter can also facilitate interventions in cases of obstructed coronary arteries. Although ICCs are an uncommon congenital variation, they can sometimes be misidentified as collateral vessels, highlighting the importance of recognizing their unique angiographic and histological characteristics. Further research is needed to fully elucidate the role of ICCs in the cardiovascular system and their potential clinical implications. Further research is needed to fully elucidate the role of ICCs in the cardiovascular system and their potential clinical implications as a single case report is not enough with the need for higher exposure and investigations, the documentation of even a single case of this nature highlights the importance of careful evaluation during coronary angiography as well as the importance of personalized patient care.
